# Occupational Influences in a Norwegian Material of 235 Cases of Primary Epithelial Lung Tumours

**DOI:** 10.1038/bjc.1954.66

**Published:** 1954-12

**Authors:** L. Kreyberg


### OCCUPATIONAL INFLUENCES IN A NORWEGIAN MATERIAL OF 235 CASES OF PRIMARY EPITHELIAL LUNG TUMOURS.

L. KREYBERG.

*From the Institutt for Generell og Eksperimentell Patologi, Universitetet i Oslo.*

Received for publication October 26, 1954.

THERE is increasing evidence to show that the greater number of lung cancer cases registered has a two-fold cause. Firstly, there is an increase as a result of better diagnostic facilities, more cases are discovered. This increase is approximately of the same order for the two sexes. Secondly, a greater number of cases, especially in males, are actually developing to-day than a few decades ago. This rise in the lung tumour frequency in males is mainly, if not exclusively, caused by squamous cell, large cell and small cell carcinomas (Group I tumours, Kreyberg, 1954*a*, 1954*b*). Further, it seems to be generally agreed that this group of tumours are exponents of one or more carcinogenic factors, and the conclusion from the collected data is that a new carcinogenic situation has been created in large parts of the world, essentially in this century, a situation which nearly exclusively hits the male. The etiologic factor(s) is (are) to be sought in the male's working conditions and/or his mode of life.

The assumption that the new carcinogenic situation consists in a general air pollution has been advocated, or accepted, by many students of the problem, mainly because of the considerably lower lung cancer frequency in rural districts, as compared to urban. This assumption meets, however, a serious objection, namely, that females, breathing the same polluted air, have not yet shown anything like the increase apparent in males.

In a recent paper the author (Kreyberg, 1954*c*) has produced figures from Norway indicating exactly the same difference between rural and urban districts, as seen in other countries. In addition he has shown that, in the smaller towns with "clean air," situated in rural districts, or in coastal areas with lively change of air, the lung cancer development closely follows the urban pattern, and that these small towns do not lag behind in the present development when compared to smaller industrial towns and centres with a more or less pollution of the air by smokes and fumes from industry. It was concluded that "the new carcinogenic situation is closely linked to the urban mode of life, and that smoke and fumes from industry are not an essential factor of that part of urban life which is responsible for the increase in lung cancer in Norway."

If a general air pollution is not of great importance, it may be nevertheless, that local dusts, smoke and fumes, to which many males especially are exposed in their work, are a causative factor. Also this assumption has been extensively discussed in the literature.

Kennaway and Kennaway (1947) examined the occurrence of lung cancer in 63 different occupations and trades for two periods, 1921–32 and 1933–38 and the reader is referred to this detailed study. The main conclusions were that : (*a*) the agriculture and coal-mining industries show a low incidence ; (*b*) a group of open-air occupations, where there is exposure to the dust of roads, has ratios above 100 for cancer of the lung . . . but the comparative incidence of cancer of the lung is not increasing distinctly in any of these occupations, and in the paviours, street masons, concretors and asphalters there has been a distinct fall in the ratio ; (*c*) the occupations in which there is a liability to silicosis do not show a high incidence of cancer of the lung ; but (*d*) cases of cancer of the lung have occurred in some occupations involving exposure to asbestos ; (*e*) no occupations involving exposure to any kind of dust, except those concerned with asbestos, arsenic and nickel ... have been found in which there might be an increased incidence of cancer of the lung ; (*f*) workers exposed to coal-gas and tar tend to show an increased prevalence of cancer of the lung, but in the later period studied the incidence does not exceed two-and-a-half times that of the general population ; (*g*) no special occupations have been found, among the 63 examined, to which the increase in the total of cases of cancer of the lung can be attributed. This increase is now so great that the incidence upon any such occupations would have to be very high indeed ; (*h*) no evidence has been found that tarring of roads has affected the incidence of cancer of the lung. Such data as are available suggest that coal-tar in the atmosphere, whether derived from roads, domestic chimneys or any other source, does not cause an exceptionally high incidence of cancer of the lung. Cotton mule-spinners show an especially small liability to cancer of the lung, although they inhale air sprayed with an oil which produces cancer of the skin. Much further work is required on the factors which regulate the penetration of particles and droplets of various shapes and sizes into the air passages.

Doll (1953), likewise from Great Britain, found that "Industrial hazards of great variety are responsible for a proportion of cases, but, with the exception of the production of gas, the industries with a recognized risk employ few workers and the total number of cases resulting each year is small," and further specifying the special risks mentions " radon and benzpyrene, products associated with the refining of nickel and the manufacture of chromates and asbestos, and probably arsenic."

Hueper in U.S.A. has dealt with this problem in a series of papers. In a paper (Hueper, 1953) he gives his opinion as follows : " Of distinctly greater significance, on the other hand, are the observations made in regard to the increased or decreased frequency of lung cancer among members of certain occupational groups, especially as these data reveal a definite degree of uniformity with which certain worker groups are cited for their excessive liability although the data are coming from different investigators and obtained from different material." And further : "While the number of respiratory cancers which have been attributed to contact with these agents is relatively small, there is good reason to believe that the actual number of occupational respiratory cancers produced by an occupational exposure with them is considerably larger."

Lickint (1953) in a German monograph on the etiology and prophylaxis of lung cancer reviews the present knowledge on industrial factors and states that " dusty work," like street work, did not play any important role. Remarkable is, on the other hand, the great number afflicted by lung cancer among office workers, merchants and males in professional work (84 out of 224 male lung cancer cases), as well as artisans, not particularly exposed to dust (66 cases).

In the following, an analysis will be made of the relationship between lung cancer and occupation, based upon a material of 235 cases comprising 8 cases from our Series I, 131 cases from Series II and 96 cases from a new Series III, representing 202 males and 33 females.

In each case the lung tumour was histologically determined and grouped according to principles previously described (Kreyberg, 1954*a*). In addition, the occupation of each patient was ascertained through a questionnaire, demanding information from decade to decade of adult life.

In Table I a survey is given of the data collected, a rather detailed differentiation of occupations being used. The patients are registered according to sex and to the histological group (Group I comprising squamous cell, large cell and small cell carcinomas ; Group II comprising adenocarcinomas bronchiolar cell carcinomas, and adenomas and salivary gland tumours of the lung).

In most cases the categorisation was simple, because the patients had followed a consistent line in their occupational development. In the cases, however, where the patient had changed occupation, an estimate was made of the individual situations, and the patient was listed under the more special occupation if such work had lasted for 10 years or more. If, for instance, a stoker of a ship had worked for 10 years or more as such and later went ashore and settled as a fisherman he would be listed as a stoker. If, on the other hand, a sailor went ashore, and had worked in a copper mine for 10 years or more he would be listed as a miner. Actually, doubts as to correct categorisation have been rare enough not to invalidate the general conclusions. This mode of approach was chosen in the hope of discovering, if any, marked accumulation of lung tumour cases in any special occupation.

Table I again indicates the even distribution of Group II tumours in the population, with the largest figures in the occupations with the largest number of men. The Group I tumours show a more irregular distribution, most conspicuous by the low number in farmers. No special occupation is overwhelmingly represented, but it may be that workers in mechanical workshops, including solderers, welders and similar workers, are over-represented. The total number, however, is small and does not permit an analysis with any accuracy. It may also be noticed that civil engineers show a comparatively high number as compared to doctors and lawyers.

In order to assess the lung cancer risks on a broader basis the different occupations have been collected in 3 main categories ; (*i*) " Open air " activities and " house "-work, (*ii*) " clerical " and " professional " work, and (*iii*) "dusty " work. When these categories of occupations had been decided upon and delimited, the Statistisk Sentralbyrå was kind enough to furnish a fairly accurate estimate of their numerical background in the population, and the data are presented in Table II.

Whereas the listing in Table I, in order to obtain certain information, had been influenced by the fact that a number of patients had at some period in their lives held a " special occupation," and the figures for the population at large give the occupation of the population at the moment of registration, the figures are not strictly comparable. The following figures may therefore be of some importance.

Out of the 202 male patients, 7 only were 70 years or more. These 7 have all been listed under their previous occupation. Further, 13 males who, for the special purpose of Table I, had been listed under another occupation than that held when the patients lung tumour was discovered, have been transferred to the latter occupation. In a series of cases this has only caused a change in occupation

TABLE I.—*Distribution of Group I and Group II Cases by Occupation.*

| Category of activities. | Occupation. | Group I. M. | Group I. F. | Group II. M. | Group II. F. |
|---|---|---|---|---|---|
| "Open air" activities and "house" work | Sailors | 13 | — | 5 | — |
| | Fishermen | 5 | — | — | — |
| | Farmers | 5 | — | 8 | 2 |
| | Timbermen, carpenters | 7 | — | — | — |
| | Gardener, park attendants | 3 | — | 1 | — |
| | Constructors | 5 | — | — | — |
| | Horse drivers | 3 | — | — | — |
| | Conductors (street car, rail) | 2 | — | — | — |
| | Patrol-policemen | 1 | — | 2 | — |
| | Telephone line workers | 1 | — | — | — |
| | Dockers | 2 | — | — | — |
| | Ships cooks | 2 | — | — | — |
| | House workers | — | 4 | — | 23 |
| | Total | 49 | 4 | 16 | 25 |
| "Clerical" and "professional" work | Office clerks | 13 | — | 3 | 3 |
| | Business men and bankers | 10 | — | 2 | — |
| | Commercial travellers | 2 | — | — | — |
| | Civil engineers | 7 | — | — | — |
| | Lawyers | 2 | — | — | — |
| | Physicians | 2 | — | — | — |
| | Dentists | 1 | — | — | — |
| | Authors | 1 | — | — | — |
| | Singers | 1 | — | — | — |
| | Hotel owners, caterers | 2 | — | — | — |
| | Storehouse superintendents | 1 | — | — | — |
| | Students | — | — | 1 | — |
| | Total | 42 | 0 | 6 | 3 |
| "Dusty" work | Firemen, stokers (land, sea) | 5 | — | — | — |
| | Engineers (land, sea) | 5 | — | — | — |
| | Locomotive drivers | 1 | — | — | — |
| | Chauffeurs | 4 | — | — | — |
| | Mechanical workers, grinders, solderers | 20 | — | 4 | — |
| | Blacksmiths | 1 | — | — | — |
| | Plumbers | 3 | — | — | — |
| | Miners, iron, copper etc. | 4 | — | — | — |
| | Stone and concrete workers | 5 | — | — | — |
| | Masons | 2 | — | — | — |
| | Brick workers | 1 | — | — | — |
| | Glass workers | 1 | — | — | — |
| | Match workers | 1 | — | — | — |
| | Painters | 4 | — | — | — |
| | Dyers, chemical factories | 1 | — | 1 | — |
| | Printers | 1 | — | — | — |
| | Shoe workers, makers | 3 | — | — | — |
| | Tailors | 3 | — | — | — |
| | Furniture makers | 4 | — | 1 | — |
| | Paper workers | 2 | — | — | 1 |
| | Textile workers | — | — | 1 | — |
| | Tar-paper workers | — | — | 1 | — |
| | Bakers | 3 | — | — | — |
| | Butchers | 1 | — | — | — |
| | Brewery workers | 1 | — | — | — |
| | Fishcanning workers | 1 | — | — | — |
| | Warehouse attendants | 2 | — | — | — |
| | Waiters | 2 | — | — | — |
| | Total | 81 | 0 | 8 | 1 |

TABLE II.—*Occupational Categories* (*Population over* 15 *years of age*, 1950).

| | | Males. | Females. |
|---|---|---|---|
| I. | (*a*) "Open air " activities : | | |
| | Agriculture, gardening, lumberwork | 263,463 | 26,587 |
| | Fishing, whaling, etc. | 69,233 | 292 |
| | Artisans and workers in building and construction | 114,601 | 822 |
| | Shipping, less office workers | 58,076 | 2,048 |
| | Transport, less office workers and less railway workers | 35,097 | 1,351 |
| | (*b*) " House " work : | | |
| | House wives, house workers, independents staying home | 504 | 822,905 |
| | | 540,974 | 854,005 |
| | Less " dusty " workers (firemen, engine personnel, painters, etc.) and waiters | 73,480 | — |
| | Total | 467,494 | 854,005 |
| II. | " Clerical " and " professional " work in : | | |
| | Business, banking, insurance administration, armed forces, civil services, etc. | 147,904 | 146,809 |
| | Mines, manufacturing, building, construction, power and water, shipping, travel, hotels, etc. | 75,763 | 26,597 |
| | | 250,667 | 173,406 |
| | Less " dusty " work in these groups | 6,504 | — |
| | Total | 244,163 | 173,406 |
| III. | " Dusty " occupations : | | |
| | Workers in mines, manufacturing, railway, gasworks, etc., and " dusty " work in the other groups. Total | 341,634 | 70,869 |
| | | | Per cent. |
| I. | " Open air " activities, " house " work : Males | 467,494 | 44·3 |
| II. | " Clerical " and " professional " work : Males | 244,163 | 23·3 |
| III. | " Dusty " occupations : Males | 341,634 | 32·4 |
| | Total | 1,053,291 | 100·0 |
| | Retired, not working | 162,518 | — |
| I. | " Open air " activities, " house " work : Females | 854,005 | 77·8 |
| II. | " Clerical " and " professional " work : Females | 173,406 | 15·8 |
| III. | " Dusty " occupation : Females | 70,869 | 6·4 |
| | Total | 1,098,280 | 100·0 |
| | Retired, not working | 164,793 | — |

and not in main occupational category. When the final correction was made, this led to a reduction in the figures for " open air " and " house "work of 3, with a corresponding increase in " clerical " work of 2, and " dusty " work of 1. These figures are now used in Table IV.

In a previous paper (Kreyberg, 1954*a*) it has been shown that the Group II tumours represent primary epithelial lung tumours not connected with any special carcinogenic situation of recent origin, whereas the Group I tumours have shown a marked increase, and a pattern of epidemiological circumstances pointing to a new carcinogenic situation.

As the females in Norway manifest nearly exclusively Group II tumours, it is of great interest, as a control, especially to examine the occurrence of such tumours in our patient material in relation to the estimated representation of the occupational categories of the population. The result is as shown in Table III.

TABLE III.—*Occurrence of Group I and Group II Tumours in Various Occupational Categories (Females).*

| Occupational category. | Estimated in the population. No. | Per cent. | Tumours occurring. Group I. No. | Group II. No. | Per cent. |
|---|---|---|---|---|---|
| "Open air" and "house" work | 854,005 | 77·8 | 4 | 25 | 86·2 |
| "Clerical" and "professional" work | 173,406 | 15·8 | — | 3 | 10·3 |
| "Dusty" work | 70,869 | 6·4 | — | 1 | 3·5 |
| Total | 1,098,280 | 100·0 | 4 | 29 | 100·0 |

Considering the limited number of cases, there is a remarkable correspondence between the numbers to be expected and the numbers actually occurring in the three main occupational categories.

Group I tumours in females occur in a negligible number, in our present material 4 cases only, but also these tumours are represented as would be expected if no special occupational hazard was involved.

In Table IV the occurrence of Group I and Group II tumours in the males is shown, likewise according to their representation in the different occupational categories.

TABLE IV.—*Occurrence of Group I and Group II Tumours in Various Occupational Categories (Males).*

| Occupational category. | Estimated in the population. No. | Per cent. | Tumours occurring. Group I. No. | Per cent. | Ratio. | Group II. No. | Per cent. | Ratio. Group I | Group II. |
|---|---|---|---|---|---|---|---|---|---|
| "Open air" and "house" work | 467,494 | 44·3 | 46 | 26·7 | 1·0 | 16 | 53·3 | 2·9 : 1 | (1) |
| "Clerical" and "professional" work | 244,163 | 23·3 | 44 | 25·6 | 1·8 | 6 | 20·0 | 7·3 : 1 | (2·6) |
| "Dusty" work | 341,634 | 32·4 | 82 | 47·7 | 2·4 | 8 | 26·7 | 10·3 : 1 | (3·6) |
| Total | 1,053,291 | 100·0 | 172 | 100·0 | — | 30 | 100·0 | | |

Again, it will be seen that there is a fair correspondence between the expected manifestation of Group II tumours and the number actually found. Turning to the Group I tumours quite another picture is seen.

As most of the Group I tumours in males are caused by special agent(s) of recent origin in certain parts of the world, and as there is no evidence in support of the assumption that any type of occupation positively protects against lung cancer, it is reasonable to accept the lowest figure recorded as an expression of the lowest exposure risk and as an indicator of the figures closest to the figures for "unavoidable" Group I tumours. As "unavoidable" Group I tumours are counted those which occurred in the population before the new carcinogenic situation was established. This lowest risk is found in the occupational category of "open air" and "house"-work. If this lowest risk is designated 1·0 (46 cases from 44·3 per cent. of the population—the nearly identical figures have nothing to do with the index), it will be seen that the risk for the category " clerical and professional " work is 1·8 and the risk for " dusty " work is 2·4.

Here the proportionate risks have been examined in relation to the calculated size of the occupational categories in the population. In order to check this observation, Table IV also gives the ratio Group I : Group II tumours. In the previous paper on geographical factors (Kreyberg, 1954*c*) this ratio has given valuable information.

If now, again, the lowest figure (2·9) is used as a basic index and designated 1·0, it will be found that the category " clerical and professional " work has the index 2·6 and the " dusty " work the index 3·6. The latter indices are of a slightly different magnitude and their significance is less than that of the first series because of the small number of Group II tumours in each occupational category. But, nevertheless, the second series of indices tends to confirm the bearing of the first, which is that there is a considerably greater chance of recording lung cancer among people occupied with " dusty " work, as compared to " open air " activities and " house "-work, and that also the category " clerical " and " professional " work shows a considerably higher index than the latter.

These findings do neither prove nor exclude that " dust," *per se*, may be of importance as a factor augmenting the chance to develop lung cancer, but two observations tend to reduce the probability of this assumption. Firstly, the considerably increased risk also among " clerical " and " professional " workers, not exposed to dust, and secondly, the wide range of occupations involved and with few victims in each occupation. Our findings so far are in accord with those of Doll (1953) and Lickint (1953), quoted above.

The conclusions of the present analysis are that industrial dusts may be of some, but cannot be of major, importance for the new carcinogenic situation created for the development of lung cancer, and cannot account for the marked increase in Group I tumours in the last few decades. The truly responsible agent, of whatever nature it might be, is to a great extent also acting on the " clerical " and " professional " workers.

In a previous paper (Kreyberg, 1954*c*) it has been shown that the increase in lung cancer (Group I cases) in Norway is intimately connected with the urban mode of life, and that a general air pollution by smoke and fumes from industry is not an important factor in that part of urban life which is responsible for the lung cancer development. This observation, together with the findings of the present study, indicate that the new carcinogenic situation is more likely linked to the males' personal life habits, than to a general or special exposure to smoke, fumes and dusts from industry, streets and motor vehicles.

### SUMMARY AND CONCLUSIONS.

A detailed analysis of 235 cases of primary epithelial lung tumours from 202 males and 33 females shows that people in " dusty " work are more liable to develop lung tumours than people with " open air " occupations and " house "-work. " Clerical " and " professional " workers likewise developed a higher number of lung tumours as compared to the category " open air " activities and " house-"-work. These observations, in addition to the previous findings, that smoke and fumes from industry in general do not seem to influence the lung tumour development, whereas urbanized living does, lead to the tentative conclusion that the new carcinogenic situation for lung cancer development, up to the present time, probably is linked to the males' personal life habits. These conclusions seem valid at the present stage of the lung cancer development in Norway.

This study has been aided by a generous grant from "Tobaksfabrikernes Landsforening av 1901".

I wish to express my sincere thanks to Dr. Knut Westlund and Dr. R. Doll for valuable advice during the preparation of this paper.

### REFERENCES.

DOLL, R.—(1953) *Brit. med. J.*, **2**, 521, 585.
HUEPER, W. C.—(1953) *R. I. med. J.*, **36**, 24.
KENNAWAY, E. L. AND KENNAWAY, N. M.—(1947) *Brit. J. Cancer*, **1**, 260.
KREYBERG, L.—(1954*a*) *Ibid.*, **8**, 199.—(1954*b*) *Ibid*, **8**, 209.—(1954*c*) *Ibid.*, **8**, 599.
LICKINT, F.—(1953) 'Ätiologie und Prophylaxe des Lungenkrebses.' Dresden und Leipzig. (Steinkopf).


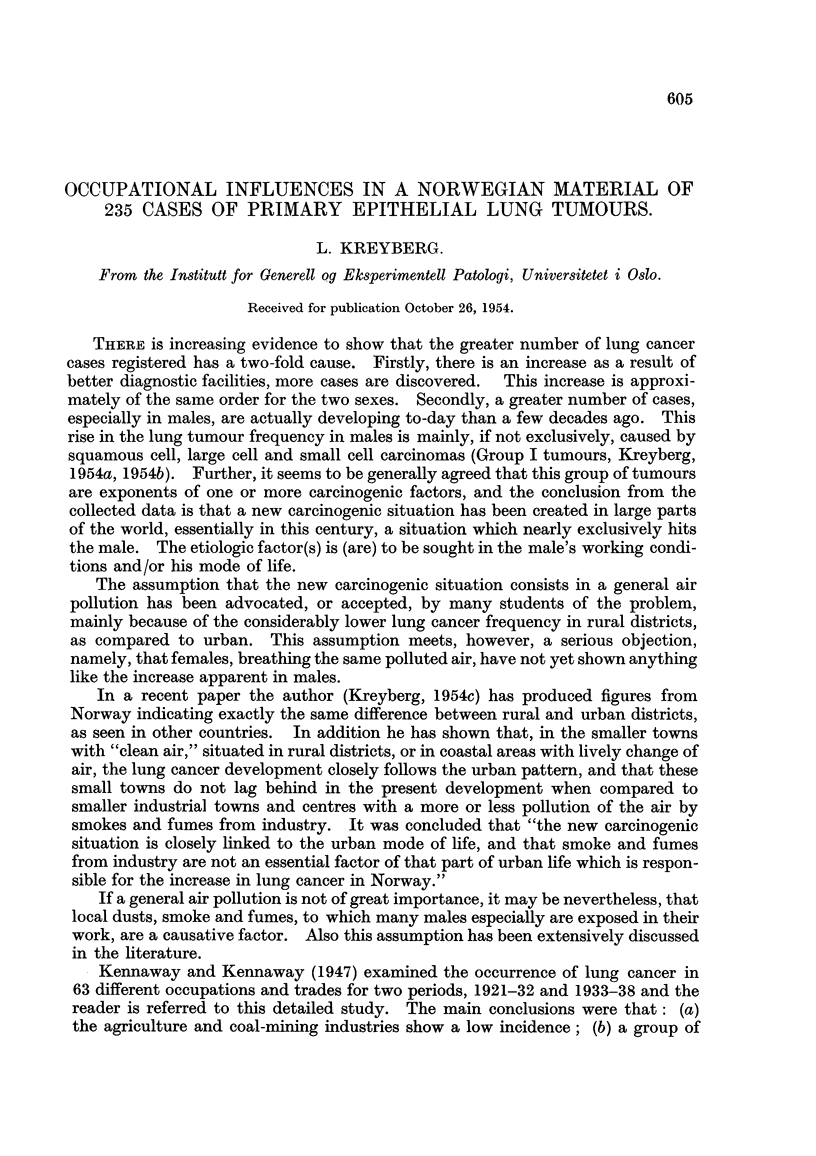

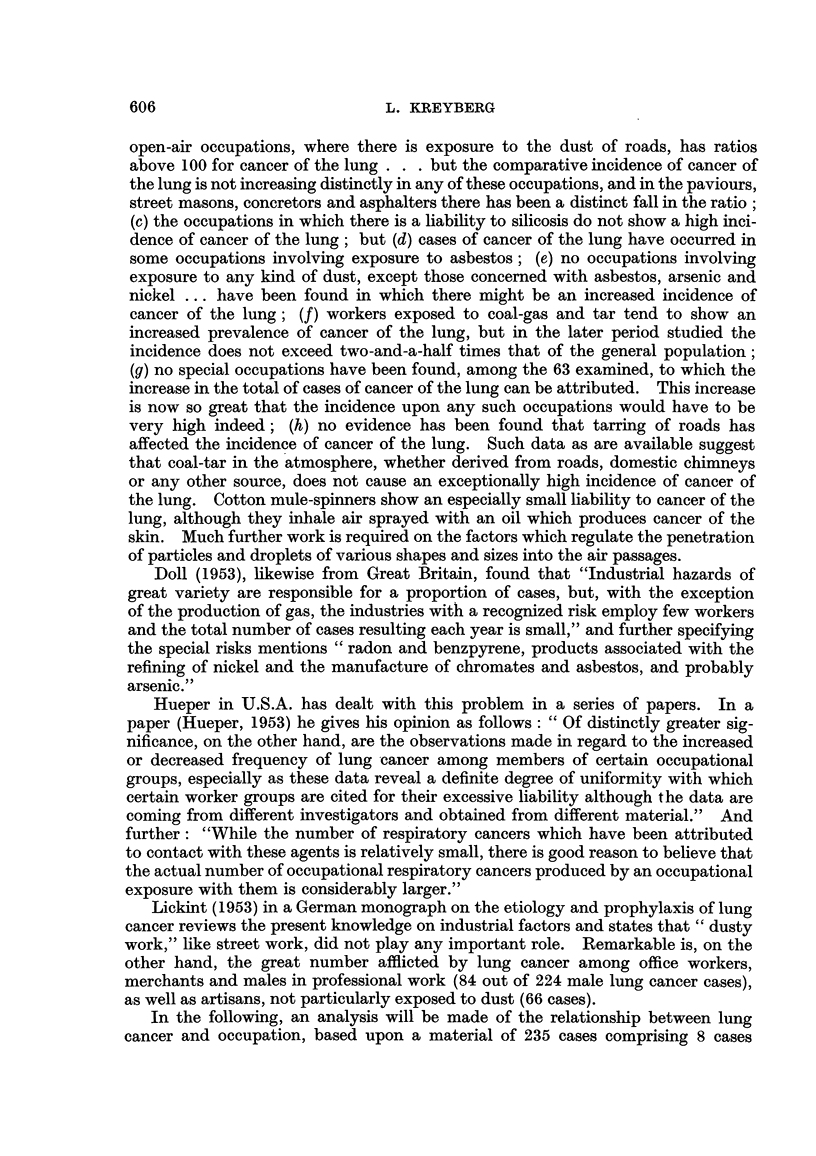

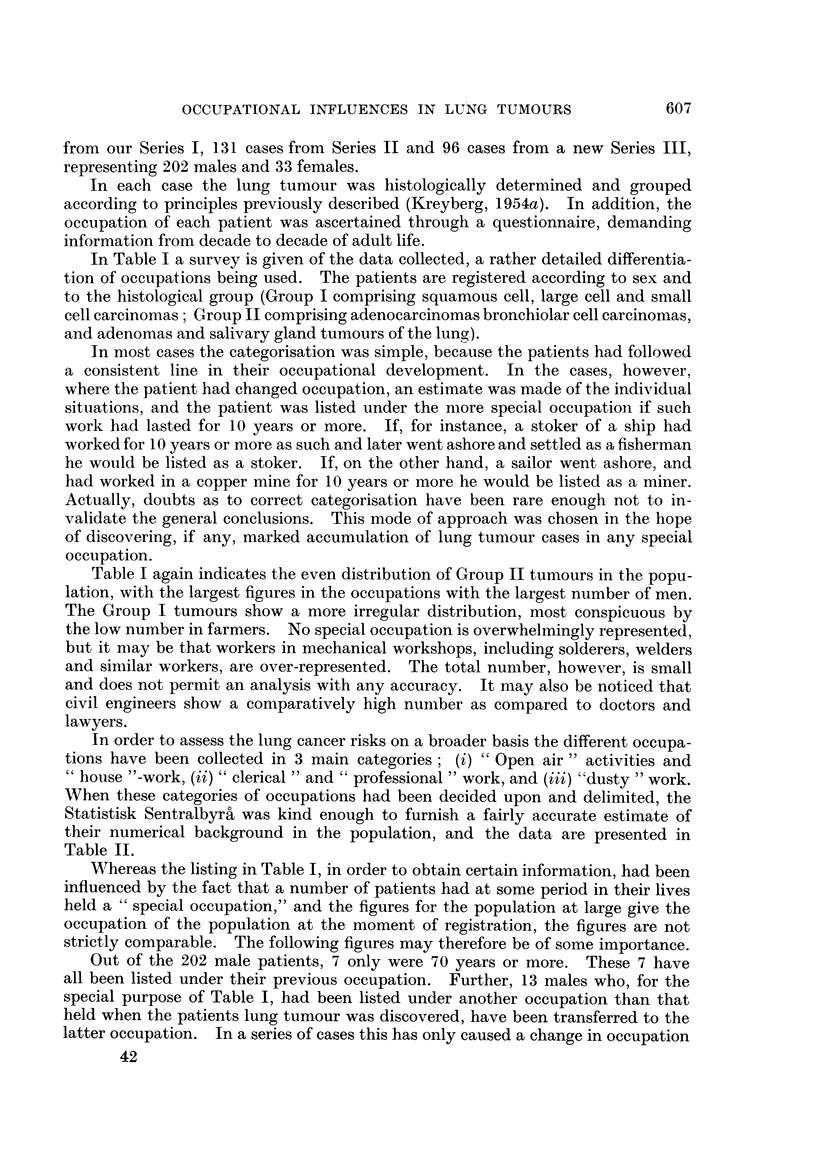

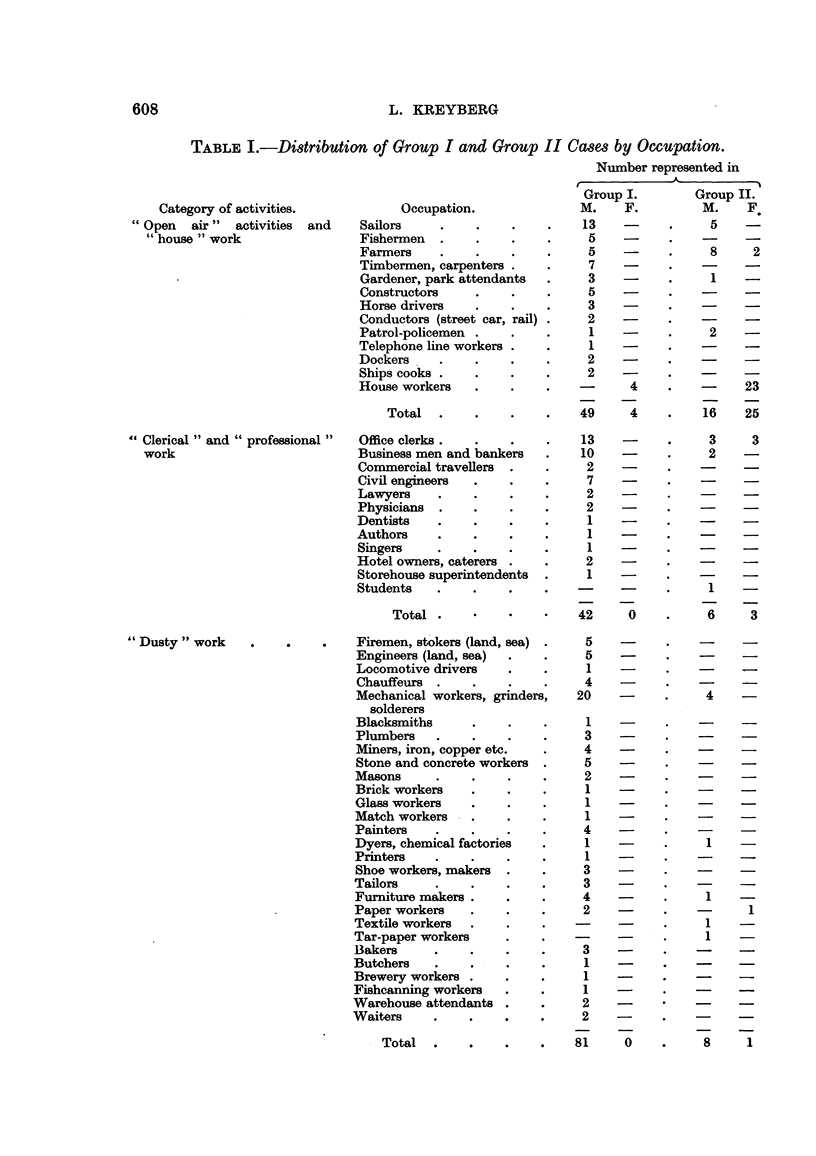

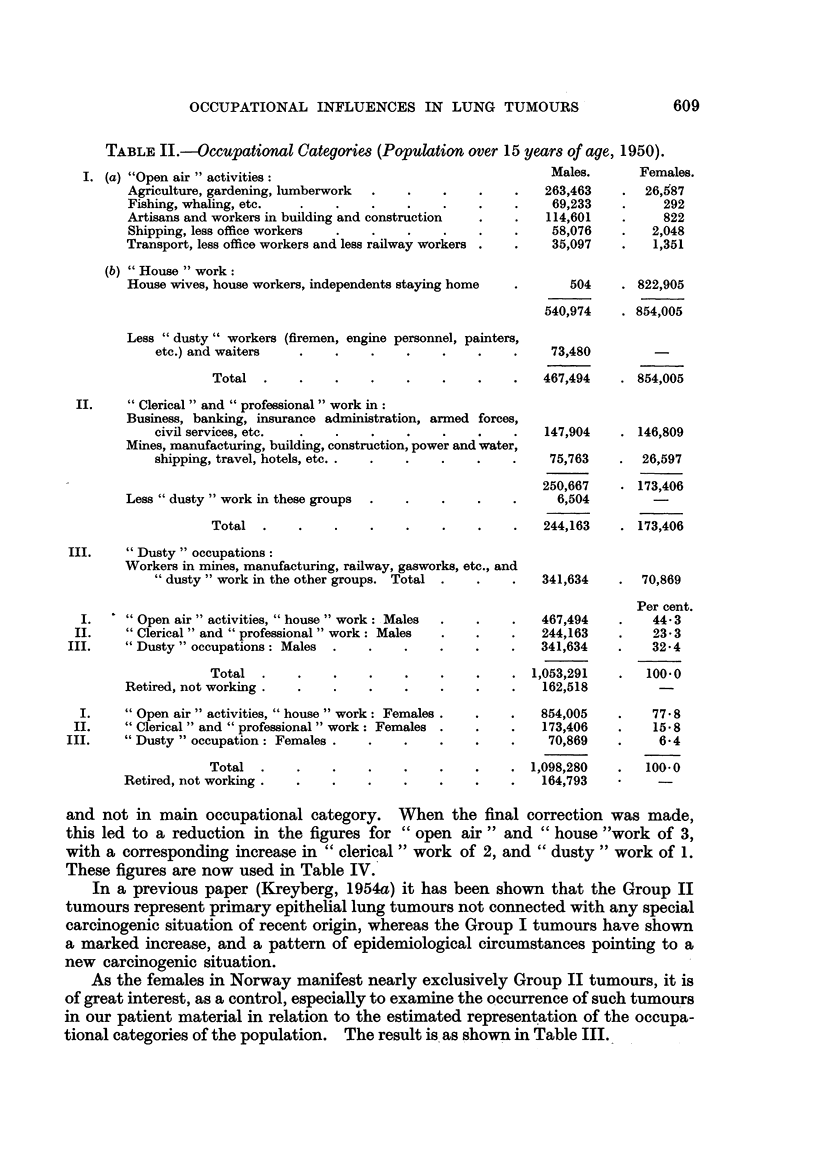

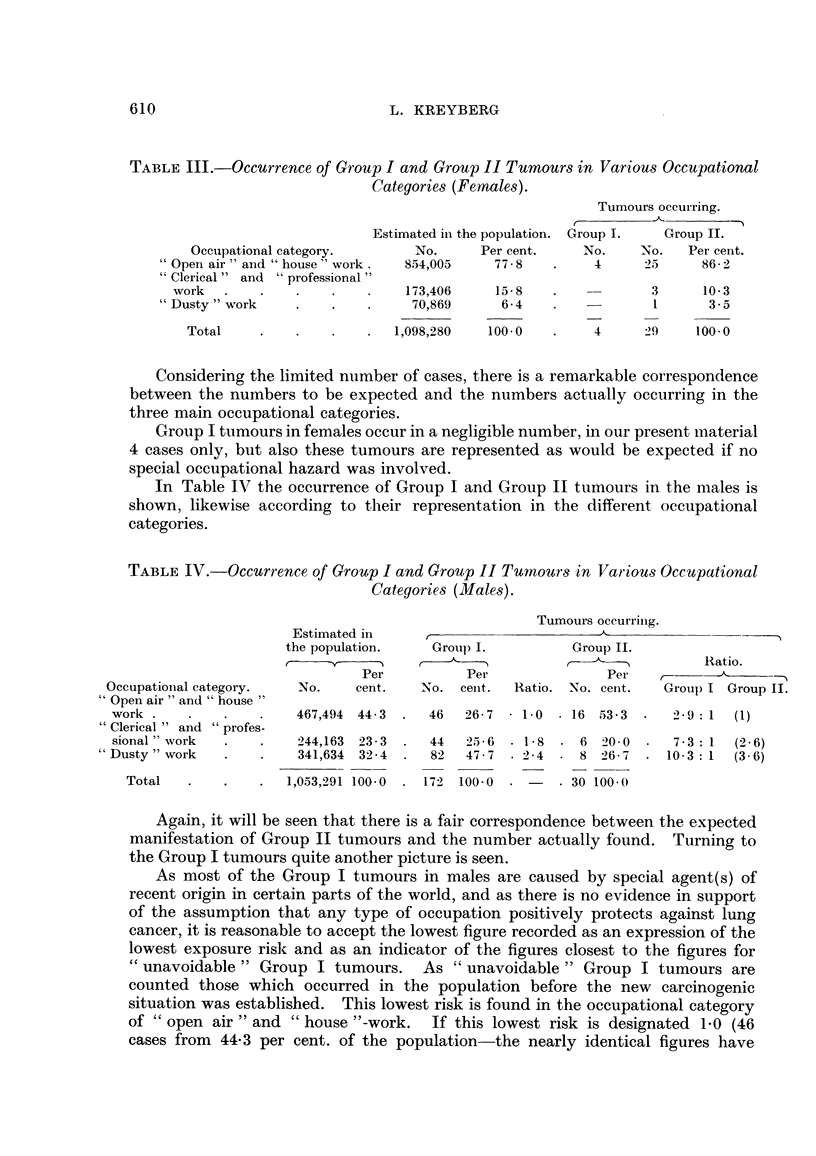

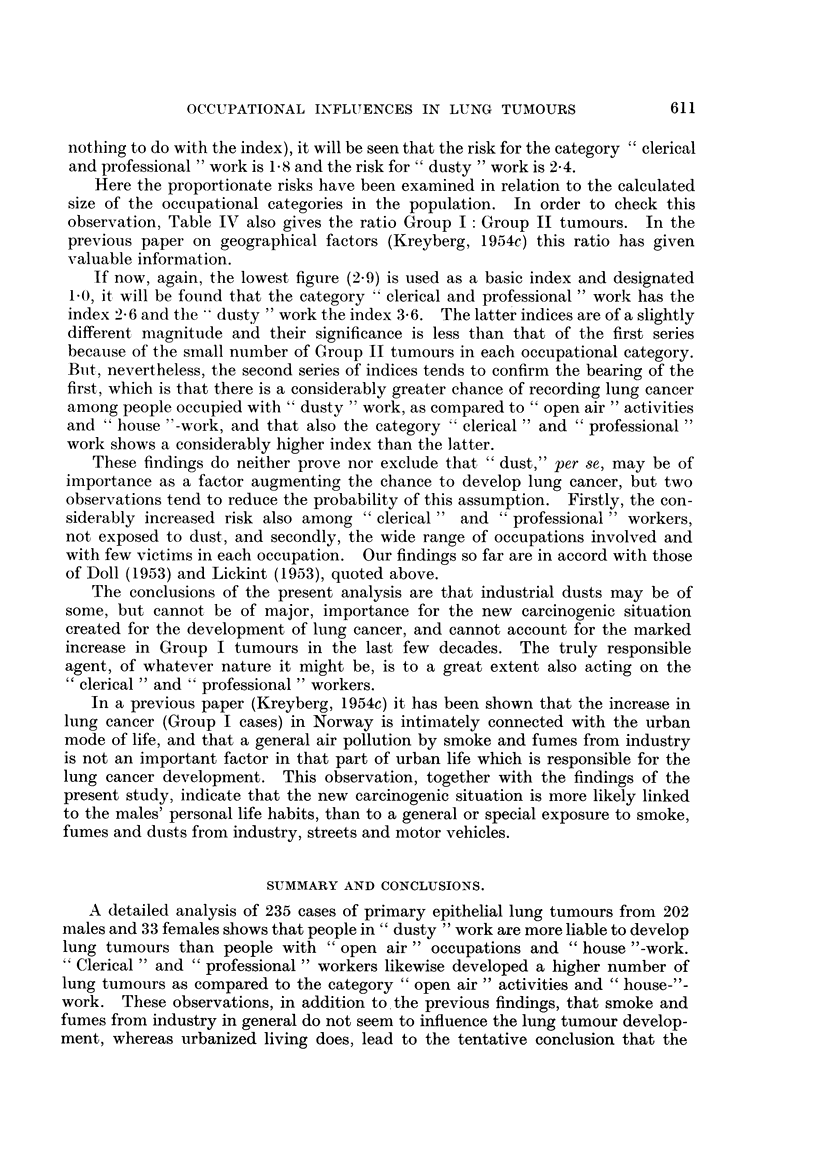

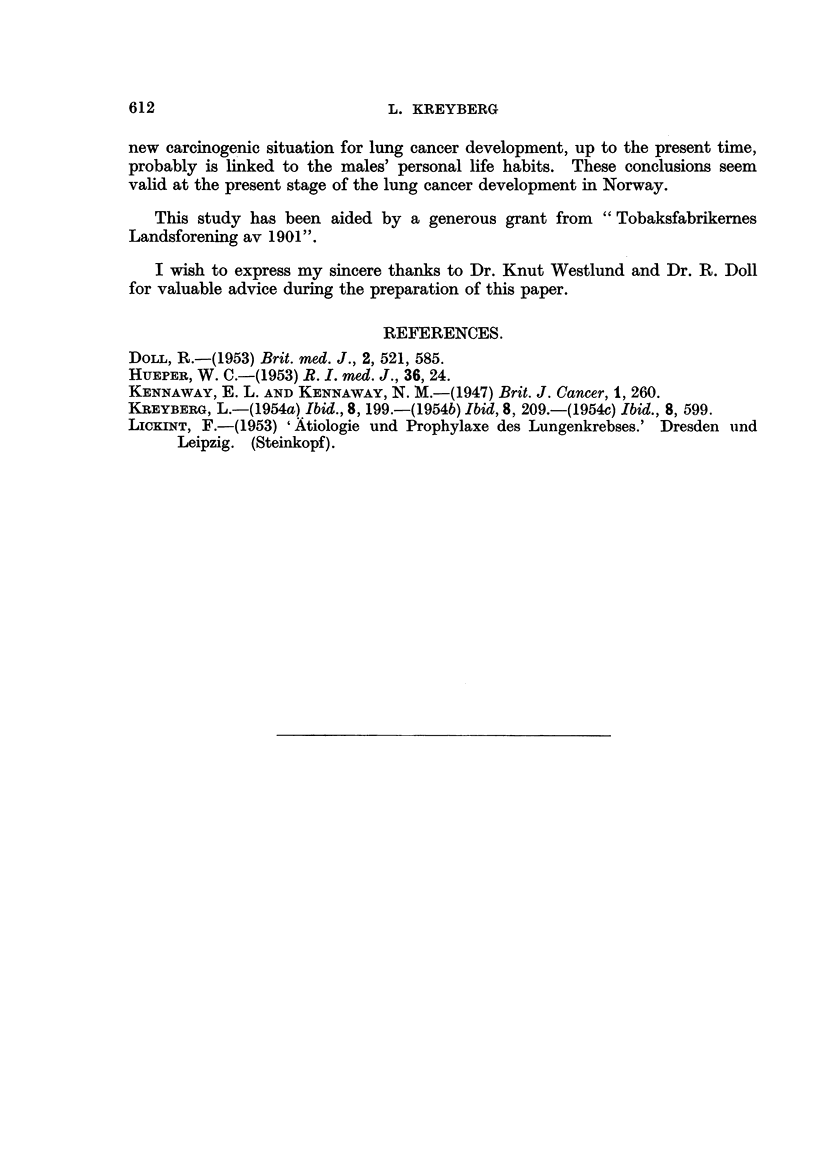


## References

[OCR_00578] DOLL R. (1953). Bronchial carcinoma: incidence and aetiology.. Br Med J.

[OCR_00584] KREYBERG L. (1954). The geographical distribution of histological sub-groups of primary epithelial lung tumours in Norway.. Br J Cancer.

